# Metachronous, non‐pineal, trilateral retinoblastoma in a patient with a seemingly reduced‐expressivity *RB1* germline deletion

**DOI:** 10.1002/ccr3.5498

**Published:** 2022-03-18

**Authors:** Saga Elise Eiset, Mikkel Funding, Hilary Racher, Steffen Heegaard, Brenda Gallie, Steen Fiil Urbak, Pernille A. Gregersen

**Affiliations:** ^1^ 11297 Department of Clinical Genetics Aarhus University Hospital Aarhus Denmark; ^2^ 11297 Department of Pediatrics and Adolescent Medicine Aarhus University Hospital Aarhus Denmark; ^3^ 11297 Department of Ophthalmology Aarhus University Hospital Aarhus Denmark; ^4^ 7938 Impact Genetics Brampton Canada; ^5^ 7938 Department of Laboratory Medicine & Pathobiology University of Toronto Brampton Canada; ^6^ Department of Ophthalmology and Pathology, Rigshospitalet University of Copenhagen Denmark; ^7^ 7938 Department of Ophthalmology The Hospital for Sick Children Toronto Canada; ^8^ 7938 Departments of Ophthalmology and Vision Science Medical Biophysics and Molecular Genetics University of Toronto Toronto Canada; ^9^ 11297 Center for Rare Disorders Pediatrics and Adolescent Medicine Aarhus University Hospital Aarhus Denmark

**Keywords:** DNA copy number analysis, intracranial tumor, RB1, retinoblastoma, trilateral

## Abstract

The clinical course of trilateral retinoblastoma can be unpredictable, and expressivity of germline *RB1* variants may vary during development. We describe an unexpected fatal case of trilateral retinoblastoma with an intracranial tumor in an unusual location and discuss genetic copy number analyses as a useful diagnostic tool with therapeutic potential.

## Graphical abstract

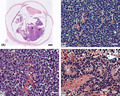

## 1 INTRODUCTION

Retinoblastoma is a rare childhood cancer of the retina (incidence 1 in 16,000 live births). In high income countries, survival rate is >95%.^1^

Retinoblastoma arises from a cell that nearly always harbors pathogenic variants in both copies of the *RB1* gene, a first and second “hit” as described by Knudson in 1971 (Knudson's two‐hit hypothesis).^2^ In non‐heritable retinoblastoma, both *RB1* variants arise in the developing retina, whereas in heritable retinoblastoma, a germline *RB1* variant is present. The majority of heritable retinoblastoma patients have a *de novo RB1* variant, as only 10% inherit the variant from a parent (autosomal dominant transmission).^2^ Patients with heritable retinoblastoma have a significantly increased risk for other primary cancers later in life, including osteosarcoma, melanoma, and pinealoblastoma.^1^, ^3^

Retinoblastoma can affect one eye (unilateral) or both eyes (bilateral). Children with bilateral disease have a pathogenic germline *RB1* variant (hence heritable retinoblastoma), but some children with heritable retinoblastoma only develop unilateral disease, due to variation in penetrance and expressivity. Trilateral retinoblastoma refers to an intracranial tumor associated with heritable retinoblastoma and occurs in 3,5% of patients with heritable retinoblastoma.^4^

In this case study of trilateral retinoblastoma, we describe a patient with an intracranial tumor in an unusual location and a challenging clinical investigation process involving extensive genetic testing.

## 2 CASE HISTORY

**FIGURE 1:**
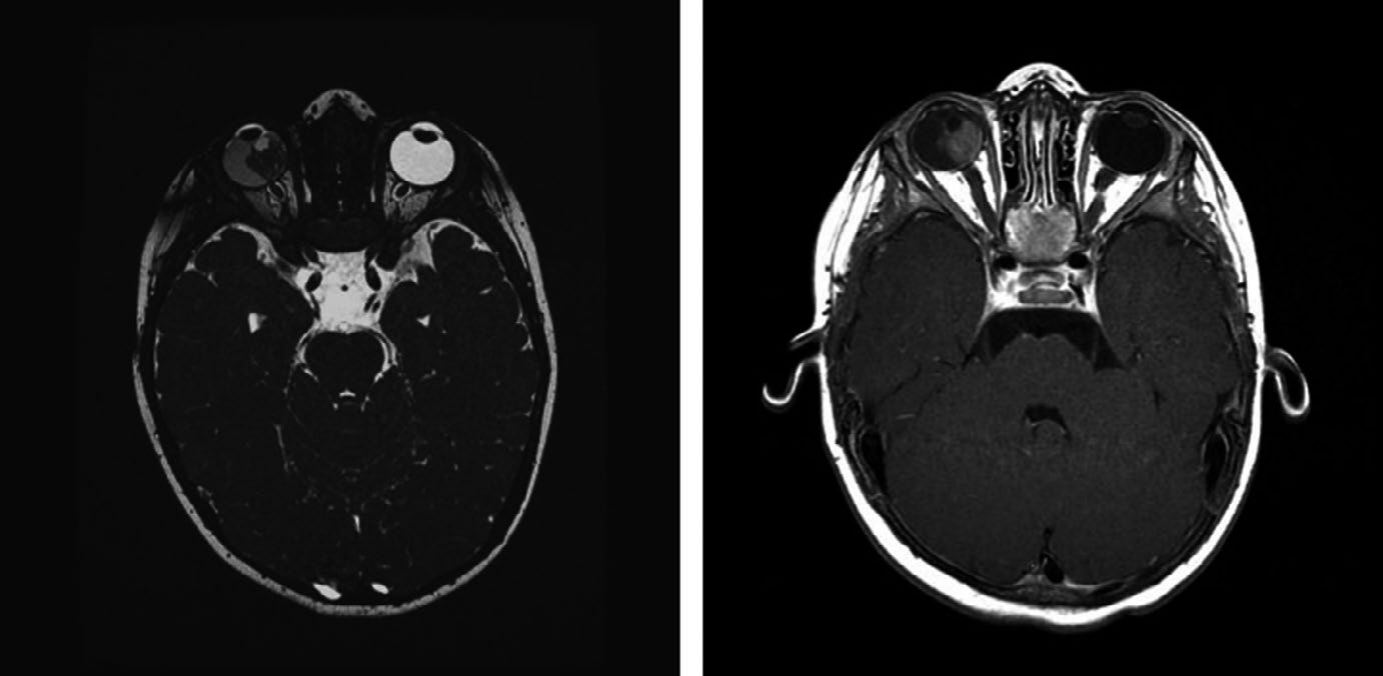
Baseline brain MRI at age 15 months

A 15‐month‐old boy presented with deviation of the right eyeball that had been slowly progressing since onset one month earlier. There was no family history of retinoblastoma, and the patient had an unremarkable prior history with normal growth and developmental milestones. The diagnosis of retinoblastoma was made on the basis of ophthalmoscopy and ultrasound of the right eye that revealed large, calcified tumor masses. The anterior chamber was normal. A baseline brain MRI was performed and revealed no extraocular extensions or trilateral disease (Figure 1).

**FIGURE 2:**
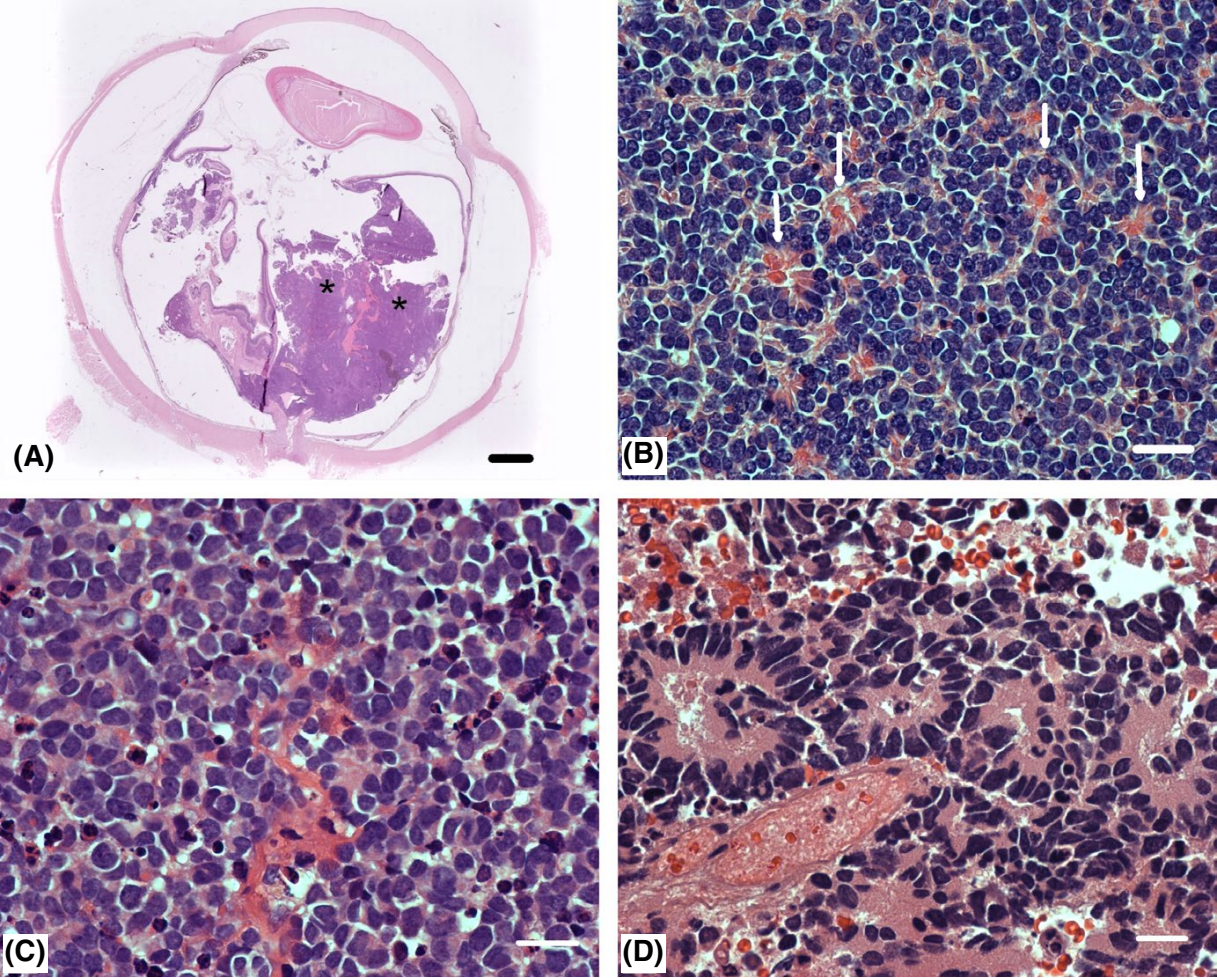
Histopathology images of the retinal (A and B) and cerebral (c and d) tumors. A. The overview of the right eye shows an intraocular tumor of the retina (asterisks) with both endo‐ and exophytic growth and with minimal invasion of the choroid and no extrascleral extension (bar = 800 μm). B. The retinal tumor cells are seen with round or oval hyperchromatic nuclei and surrounded by a scant amount of cytoplasm. Homer‐Right rosettes are seen (white arrows): The tumor is consistent with a retinoblastoma (bar = 40 μm). C. The cerebral tumor shows poorly differentiated round tumor cells with hyperchromatic nuclei. In general, the tumor shows a diffuse growth pattern (bar = 40 μm). D. In small areas of the cerebral tumor, rosettes are also seen (bar = 40 μm). The cerebral tumor is consistent with a retinoblastoma

The eye was staged Group E according to International Intraocular Retinoblastoma Classification,^5^ cT3 TNMH.^6^ Pathology showed retinoblastoma with minimal invasion of the choroid, and no extrascleral tumor cells, invasion of the optic nerve, or tumor cells in the anterior chamber, pT2a (Figure 2).^6^ The patient was treated according to guidelines with surgical enucleation of the right eye, without adjuvant chemo‐ or radiation therapy.^7^ Because of the risk of contralateral retinoblastoma, the patient was monitored with retinal examination under anesthesia every 4 weeks.

A germline deletion encompassing exon 2 and at least part of exon 1 of *RB1* was detected on DNA extracted from blood. The deletion was not detected in DNA from blood from either of the parents. However, mosaicism in either parent could not be ruled out, and the couple was offered prenatal testing in the future pregnancies.

Almost 2 years after the primary diagnosis, at age 37 months, the patient was admitted to a local pediatric center with a left side peripheral facial nerve palsy and vomiting. In the 8 weeks prior to admission, the patient had experienced hoarseness, fatigue, weight loss, and a dry cough that did not respond to antibiotic treatment. The parents had noticed a lump on the left side of the neck 3 weeks prior to admission, which had been growing.

**FIGURE 3:**
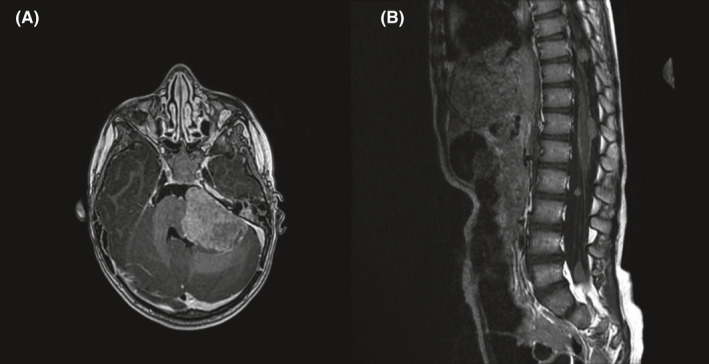
Trilateral retinoblastoma diagnosed at age 37 months. MRI shows (A) a large tumor (3.5 × 5.5 × 5.5 cm) in the cerebellopontine angle and subependymal tumor elements in the rim of the lateral ventricle anterior horns and (B) several drop metastases in the thoracolumbar spinal canal

The patient was transferred to a specialized children's oncology unit. MRI of the neuroaxis revealed a large tumor (3.5 × 5.5 × 5.5 cm) in the cerebellopontine angle, subependymal tumor elements in the rim of the lateral ventricle anterior horns, several drop metastases in the thoracolumbar spinal canal, and enlarged lymph nodes on the left side of the neck (Figure 3). There were no signs of tumor masses in the left eye or the corpus pineale.

A surgical decompression with partial resection of the cerebral tumor was performed, and pathology review confirmed the diagnosis of a malignant neuroblastic tumor that could represent either a medulloblastoma or a retinoblastoma, the latter being the most likely diagnosis due to the patient history. The patient was treated with eight cycles of intravenous and intrathecal chemotherapy, followed by high‐dose chemotherapy and stem cell reinfusion. Brain MRIs showed regression throughout the treatment, and a supplemental positron emission tomography (PET) scan performed at one‐month follow‐up showed no pathological activity in residual lesions in the left cerebellopontine angle and the spinal canal. The three‐month follow‐up brain and spine MRI showed no changes.

Five months after treatment was completed, the patient was admitted with sudden‐onset vomiting, headache, fatigue, and loss of balance. MRI showed multiple infra‐ and supratentorial processes bilaterally, and several small nodules in the ventricles. Aspergillosis was suspected, but biopsy showed malignant cells that were confirmed to be retinoblastoma (Figure 2). The patient received palliative care and died 2 weeks later, at 50 months of age.

## 3 MOLECULAR GENETIC INVESTIGATIONS

A heterozygous deletion of exon 2, and at least part of exon 1, of *RB1* was detected in DNA from the patient's blood. Quantitative multiplex PCR (QM‐PCR) confirmed that only one copy of exon 2 was present. For exon 1, three sets of non‐overlapping primers showed only one copy, the first primer being located at the position NM_000321.2(*RB1*): c.101. Analysis of the *RB1* promoter and exon 3 revealed 2 copies.

It was suggested that the deletion might be a reduced‐expressivity variant, as the patient was only unilaterally affected at the time, and it is known that some exon 1 variants show reduced penetrance due to utilization of alternative transcription start sites.^8^, ^9^ The germline deletion was also detected at a heterozygous allele frequency in the patient's primary, intraocular tumor; a second *RB1* pathogenic variant was not identified after sequencing all *RB1* coding sequence and flanking noncoding regions, methylation‐sensitive PCR analysis of the *RB1* promoter,^10^ and copy number analysis by multiplex ligation‐depended probe amplification (MLPA, SALSA P047‐D1 RB1 MRC‐Holland, Amsterdam, The Netherlands). To confirm presence of tumor DNA in the intraocular tumor sample, analysis for well‐recognized retinoblastoma somatic copy number changes in the genes *KIF14*, *DEK*, *E2F3*, *CDH11*, and *MYCN* was performed using QM‐PCR.^11^, ^12^ The analysis showed three copies of *DEK* and four copies of *MYCN*, confirming the presence of tumor DNA.

When the patient was diagnosed with an intracranial tumor, further genetic analyses were performed to help determine whether the tumor was a primary tumor (trilateral retinoblastoma) or a metastasis. MLPA analysis of the intracranial tumor identified homozygous loss of *RB1* exons 1–2 (Table 1) as well as a gain of *RB1* exons 3–27. This result potentially suggests that the intracranial tumor DNA may have developed by loss of the normal *RB1* allele and two rounds of endoreduplication of the abnormal copy (loss of heterozygosity, LOH). LOH is frequently the second mutational event leading to the development of a tumor in both retinoblastomas as well as in other cancers.^13^ Analysis for somatic copy number changes in the *KIF14*, *DEK*, *E2F3*, *CDH11*, and *MYCN* genes showed differences in comparison with the intraocular tumor (Table 2), suggesting that the intraocular and the intracranial tumors were independent in origin, predisposed by the presence of the germline *RB1* variant.

**TABLE 1:** Comparison of genetic test results

Sample	*RB1* allele 1	*RB1* allele 2
Intracranial tumor	del1‐>2	del1‐>2
Eye tumor	del1‐>2	*Normal*
Blood	del1‐>2	*Normal*

**TABLE 2:** Genetic copy number analysis of the patient's tumors

Tumor sample	Copy numbers				
	*KIF14*	*DEK*	*E2F3*	*CDH11*	*MYCN*
Intracranial	5	6	5	2	5
Eye	2	3	2	2	4

## 4 DISCUSSION

We describe a clinical case of trilateral retinoblastoma that is atypical in several aspects. Non‐pineal tumors in trilateral retinoblastoma usually occur synchronously, and almost exclusively in the supra‐ or parasellar regions.^14^, ^15^, ^16^ Accordingly, we have identified only two previous cases with a non‐pineal tumor not located to the supra‐ or parasellar regions.^17^, ^18^ As described in our presented case, the tumors were instead located in relation to the fourth ventricle and cerebellum. In both previous cases, however, the patients had been treated for bilateral retinoblastoma prior to the diagnosis of metachronous, trilateral retinoblastoma. The first case, presented by Finelli et al. in 1995, describes a girl with a family history of retinoblastoma, who was diagnosed with bilateral retinoblastoma at age 7 weeks, at which point a brain CT was normal. At age five months, she had developed bilateral recurrent ocular disease and an MRI of the brain showed a mass in the fourth ventricle, extending from the inferior vermian region along the right cerebellar hemisphere. Histopathology showed a primitive neuroectodermal tumor with neuronal differentiation.^17^ The second case, presented by Elias et al. in 2001, describes a girl who was diagnosed with retinoblastoma of the right eye at age 9 months, and a second primary neoplasm of the left eye one month later. MRI of the brain was normal. Cytogenetic investigations revealed a germline deletion on chromosome 13 with breakpoints at q12.3 and q21.3, thus encompassing the *RB1* gene that originated from a balanced chromosomal rearrangement in the mother. At age 4 years, the patient had no sign of recurrent eye disease, but had developed a large, symptomatic tumor located to the midline cerebellum. She died months later from disseminated disease with spinal involvement. Autopsy and thorough histopathological assessment were performed, and the cerebellar tumor was found to be a medulloblastoma that originated separately from the ocular tumors, based on histomorphological and immunocytochemical features.^18^ In our presented case, the same conclusion was reached by supplement of genetic copy number analysis, to confirm the diagnosis of trilateral retinoblastoma with a primary tumor located to the cerebellopontine angle.

In addition to being a diagnostic tool, characterization of somatic alterations is the basis of personalized oncology therapies, developed to improve treatment outcomes and minimize long‐term sequelae by selectively targeting cancer cells. For some cancer types, targeted treatments are well established, such as the use of tyrosine kinase inhibitors in Philadelphia chromosome‐positive leukemia^19^ and the use of PARP inhibitors in ovarian‐ and breast cancers harboring somatic or germline pathogenic variants in certain genes, including *BRCA1* and *BRCA2*.^20^ Pediatric cancer tumors in general—and retinoblastomas in particular—harbor less somatic genomic alterations than cancerous tumors in adults, but the potential for targeted treatments is promising.^19^, ^21^, ^22^ Among possible targets being studied in retinoblastoma are *E2F3* and *MYCN*,^23^ which were amplified in the tumors of the patient described in this case report.

More than 50% of trilateral retinoblastomas can be diagnosed by a baseline brain MRI in relation to the primary diagnosis of retinoblastoma,^4^ and this is now generally accepted to be the standard approach.^7^, ^24^ Whether further screening for trilateral retinoblastoma should be implemented remains unsolved. In a recent meta‐analysis by De Jong et al., it is estimated that if a brain MRI screening was implemented, with scans every six months from diagnosis to age 36 months, it would take at least 311 scans to detect one asymptomatic pineal trilateral retinoblastoma and 776 scans to save a single life.^24^ The authors also found that there is no association between age at diagnosis of intraocular‐ and pineal retinoblastoma, or between the laterality of intraocular retinoblastoma and the age at diagnosis of pineal trilateral retinoblastoma. They suggested that their findings could be due to an independent development of intraocular and pineal retinoblastoma, and that penetrance and expressivity of the germline *RB1* variant may vary during development.

Similarly, in a review by Yamanaka et al., no difference in latency period between intraocular and tertiary retinoblastoma of any location was found, when comparing patients according to laterality of their intraocular retinoblastoma.^15^ In line with these findings, our report of a patient with non‐pineal trilateral retinoblastoma demonstrates an apparent variation in expressivity. The patient presented with unilateral and localized intraocular disease, was found to carry a seemingly reduced‐expressivity *RB1* germline variant, and was expected to have a good prognosis. However, the patient developed aggressive tertiary disease that initially responded to treatment but recurred shortly after.

In conclusion, the clinical course of trilateral retinoblastoma can be unpredictable. Whether screening for trilateral retinoblastoma should be implemented is still a subject of debate, and no international consensus exists. Genetic analysis can be a useful diagnostic tool in challenging clinical investigations of trilateral retinoblastoma and has therapeutic potential.

## CONFLICTS OF INTEREST

The authors declare no conflicts of interest.

## AUTHOR CONTRIBUTIONS

Saga Elise Eiset and Pernille Axél Gregersen drafted the manuscript. Mikkel Funding, Hilary Racher, Steffen Heegaard, Brenda Gallie, and Steen F. Urbak contributed to relevant parts of the manuscript. All authors reviewed and approved the final manuscript.

## ETHICAL APPROVAL

None.

## CONSENT

Informed consent was obtained from both of the patient's parents for publication of this case report and clinical images. Written documentation of consent in accordance with the institution's patient consent policy is available upon request.

## Data Availability

None.
